# Comparative Efficacy of Percutaneous Laser Disc Decompression (PLDD) and Conservative Therapy for Lumbar Disc Herniation: A Retrospective, Observational, Single-Center Study

**DOI:** 10.3390/jcm14124235

**Published:** 2025-06-14

**Authors:** Domenico Policicchio, Benedetta Boniferro, Erica Lo Turco, Giuseppe Mauro, Antonio Veraldi, Virginia Vescio, Giuseppe Vescio, Giosuè Dipellegrini

**Affiliations:** 1 Department of Neurosurgery, Azienda Ospedaliero Universitaria “Renato Dulbecco” di Catanzaro, 88100 Catanzaro, Italy; bboniferro@aocz.it (B.B.); loturco.erica@gmail.com (E.L.T.); gmauro@aocz.it (G.M.); veraldizep@libero.it (A.V.); gvescio@aocz.it (G.V.); 2 Department of Radiology, Azienda Ospedaliero Universitaria “Renato Dulbecco” di Catanzaro, 88100 Catanzaro, Italy; vvescio@aocz.it; 3 Department of Neurosurgery, Azienda Ospedaliero Universitaria di Sassari, 07100 Sassari, Italy; giosuedipellegrini@gmail.com

**Keywords:** lumbar disc herniation, percutaneous laser disc decompression, minimally invasive spine surgery, low back pain, pain management

## Abstract

**Background:** Although percutaneous laser disc decompression (PLDD) has been proposed as an alternative to conventional surgery for lumbar disc herniation (LDH), we specifically propose it for patients with contained herniations where standard surgical intervention is not the first option. This study evaluates PLDD compared to conservative therapy as an early treatment alternative. **Methods**: This retrospective observational study compared PLDD to conservative treatment in adult patients with contained LDH. All patients underwent 3 months of standard conservative therapy. Those who remained dissatisfied according to the Visual Analog Scale (VAS) and/or Macnab criteria were then treated with PLDD. We analyzed outcomes from both treatment phases using the Wilcoxon signed-rank test and the Mann–Whitney U test. **Results**: 121 patients underwent outpatient evaluation for LDH and received an average of 90 days of conservative therapy. Of these 103 patients, dissatisfied with the outcomes of conservative treatment, subsequently underwent PLDD. Following conservative treatment, the average VAS score reduction was 4.1%. Six months after PLDD, the VAS scores demonstrated a significant reduction, with an average decrease of 30% (*p* < 0.0001). In terms of functional outcomes assessed by the Macnab criteria, 39.8% of patients treated with PLDD achieved ‘Excellent’ or ‘Good’ outcomes, compared to only 11.4% after conservative treatment. **Conclusions**: PLDD appears to be a viable alternative to conservative therapy for this subgroup of patients with contained LDH. It may be beneficial to propose PLDD early in the therapeutic regimen to accelerate short term clinical improvement. Further studies are required to evaluate the long term efficacy of this treatment approach.

## 1. Introduction

Lumbar disc herniation (LDH) is a globally prevalent condition affecting the adult population, with an incidence rate of 2–3% [1,2]. While the majority of patients recover with conservative treatment (including medications and physical therapy), a subset of patients endures debilitating low back and radicular pain that severely affects their quality of life. Standard surgical treatment (like microdiscectomy) for LDH becomes necessary in cases of failed conservative treatment with unrelenting, severe pain or neurologic deficit. Although short term pain control outcomes appear to be better with microdiscectomy, long term outcomes between surgical and conservative groups are similar [1,3]; moreover, surgery entails a greater risk of potential complications compared to conservative methods, and not all individuals are suitable candidates for microdiscectomy, especially in cases involving small, contained herniations, which are associated with less favorable surgical outcomes [4,5].

In light of these considerations, minimally invasive percutaneous treatments have been introduced with encouraging outcomes and could be considered a halfway point between conservative and surgical therapy [3]. Among these, percutaneous laser disc decompression (PLDD) stands out as a minimally invasive technique that is regarded as safe and effective for a subset of patients. However, literature reports are conflicting regarding its actual efficacy and appropriate therapeutic indications, particularly when considering its use as an alternative to either standard conservative therapy or as a substitute for surgery. Excluding patients with clear indications for microsurgical treatment as per established guidelines (e.g., progressive significant weakness of the lower extremities and/or cauda equina syndrome), several authors have suggested PLDD as a valid alternative, particularly for patients with contained disc herniations. Nevertheless, there remains a lack of consensus in the literature regarding its effectiveness [6,7,8,9].

In our center, the primary indication for PLDD was persistent radicular pain from non-extruded disc herniation lasting over 3 months and unresponsive to conservative treatment. In the absence of neurological deficits, we consider PLDD to be an alternative to conservative therapy rather than to standard surgical treatment.

The aim of our study is to compare the efficacy of standard conservative therapy (medical and physical therapy) to PLDD in the treatment of contained lumbar disc herniation in an adult population. Therefore, we retrospectively analyzed a historical cohort treated at our center, evaluating outcomes after conservative treatment and following PLDD intervention.

This research intends to contribute to the ongoing discourse by providing a comparative analysis of conservative and minimally invasive interventions, offering insight into potential pathways for patient-specific treatment strategies informed by both clinical outcomes and the current body of evidence.

## 2. Materials and Methods

### 2.1. Study Design

This retrospective observational single-cohort study was reported according to the STROBE guideline; ethical review and approval were waived for this study due to the retrospective observational nature of the study and the use of standardized treatments already approved by practical guidelines, which did not require any deviation from routine clinical practice.

The study aimed to assess the efficacy of percutaneous laser disc decompression (PLDD) against standard conservative therapy in treating adult patients with lumbar disc herniation.

Inclusion Criteria:

- Patients suffering from lumbar disc herniation with radicular pain
- Age more than 18 years
- Alignment between the side of the herniation (identified by MRI) and the side of the pain
- Informed consent was obtained

Exclusion Criteria:

- Presence of sequestered disc herniations
- Severe spinal canal stenosis
- Cauda equina syndrome
- Need for surgical fusion due to instability
- Disc infections or spinal tumors
- Pregnancy
- General contraindications to surgery

**Figure 1:**
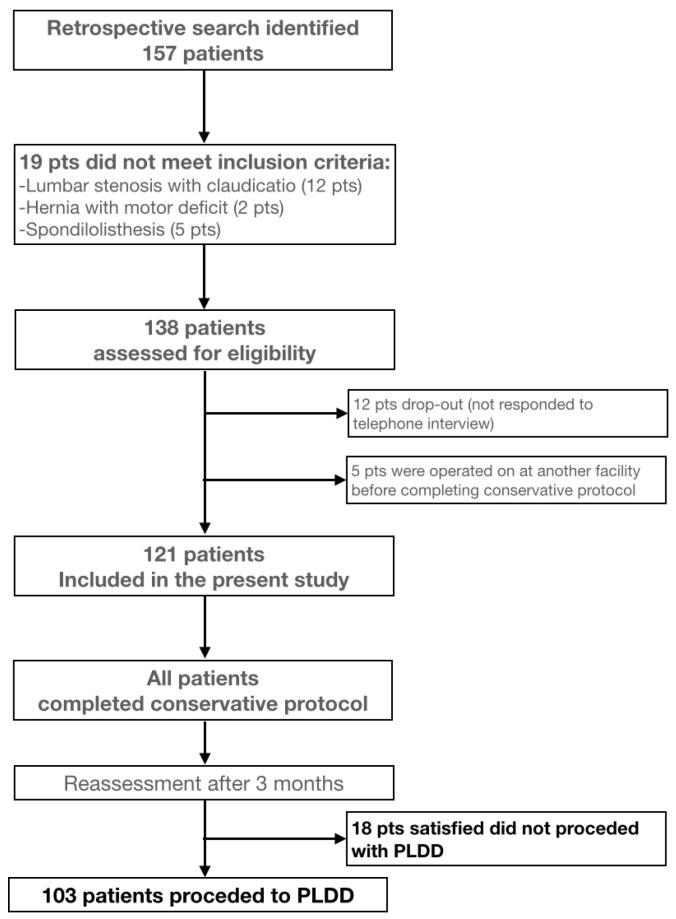
Flowchart of patient selection. A total of 157 patients were retrospectively reviewed. Nineteen patients were excluded due to conditions not meeting inclusion criteria. Of the remaining 138, 12 were considered dropouts and 5 were operated on at another hospital.

Figure 1 illustrates the patient selection process, including the application of inclusion and exclusion criteria, drop-out cases, and final cohort allocation. Upon outpatient selection, individuals meeting the inclusion criteria were offered PLDD surgical treatment. Accepting patients were enrolled on a waiting list (average wait time of 90 days) and began a regimen of standard medical therapy including a short course of NSAIDs, pregabalin, acetaminophen, B-group vitamins, and l-acetylcarnitine, supplemented by physical therapy. Prior to their surgical admission, a reevaluation was conducted using the Visual Analog Scale (VAS 0–10) for pain assessment and Macnab criteria (Excellent, Good, Fair, Poor). Patients who reported significant improvement were excluded from surgery, while those with no significant improvement advanced to the PLDD intervention.

This identified two distinct phases of treatment: conservative treatment (all patients placed on the waiting list and subjected to standard conservative therapy, irrespective of their subsequent choice regarding surgery) and PLDD surgical treatment (patients who, after the standard conservative therapy, elected to proceed with PLDD surgical treatment due to unsatisfactory pain relief).

### 2.2. Data Collection

Data were collected retrospectively via telephone interviews and by reviewing waiting and surgical lists to identify patients who refrained from surgery following positive outcomes from medical therapy. This method allowed for a direct comparison between outcomes from conservative therapy alone and those from subsequent PLDD surgery, based on VAS and Macnab criteria.

### 2.3. PLDD Surgical Procedure

The procedure is conducted under local anesthesia with the patient in a prone position. We employ a freehand technique, guided by bidimensional fluoroscopy, to insert a percutaneous laser probe into the disc. Details of the surgical technique are extensively described elsewhere. We utilize the Laser System Velas II 30B (650 nm diode laser, power < 5 mW) equipped with laser fibers arch discharged featuring spherical diffusion (produced by Nanjing Chunhui Science and Technology Industrial Co., Ltd. (Nanjing, China), distributed by AGC Orthopaedics Srl). Initially, the probe is inserted into the center of the disc, where it releases 250 joules of energy over 20 s. It is then repositioned to the posterolateral edge on the herniated side, releasing an additional 250 joules in another 20 s, thereby achieving vaporization of the disc material under real-time fluoroscopic monitoring.

### 2.4. Postoperative Care

Following surgery, patients received a three-day course of corticosteroid therapy (betamethasone 4 mg/day) and continued with pregabalin, l-acetylcarnitine, and vitamin B12 for two weeks, and acetaminophen as needed.

### 2.5. Follow-Up

All patients were clinically evaluated at 15 and 30 days post-discharge through scheduled outpatient visits. Additionally, a structured telephone interview was conducted between December 2023 and February 2024 by a physician not involved in the surgical management. Patients were asked to retrospectively report their outcomes—including pain levels (VAS) and functional status (Macnab criteria)—during the first six months after the PLDD procedure, allowing for a standardized six-month follow-up period across the entire cohort.

### 2.6. Outcome Measures

The assessment focused on evaluating pain reduction (VAS score 0–10) and functional improvement (Macnab criteria) at 6 months along with rates of complications.

The Macnab criteria are defined as follows: Excellent: no pain, no restriction of mobility, return to normal work and level of activity; Good: occasional non-radicular pain, relief of presenting symptoms, able to return to modified work; Fair: some improved functional capacity but still handicapped and/or unemployed; Poor: continued objective symptoms of root involvement; additional operative intervention needed at the index level irrespective of length of postoperative follow-up.

### 2.7. Data Analysis

This encompassed outcome comparisons before and after each treatment (conservative vs. PLDD), deploying descriptive statistics for baseline characteristics, along with subgroup analysis. Evaluations of safety and complication rates thoroughly assessed the efficacy and associated risks of the procedures. For the analysis of results, statistical tests, including the Wilcoxon signed-rank test and the Mann–Whitney U test, will be employed to compare changes within and between the treatments, respectively. These tests were selected due to their robustness in handling non-parametric data, often found in clinical research involving pain scores. A *p*-value of less than 0.05 was considered statistically significant for all tests.

## 3. Results

A total of 157 patients were retrospectively reviewed. 19 were excluded due to conditions not meeting inclusion criteria (lumbar stenosis with claudication in 12 patients, extruded hernia causing motor deficit in 2 patients, and unstable spondylolisthesis in 5 patients). The dataset included 138 patients listed for PLDD surgery to treat lumbar disc herniation from May 2022 to July 2023. Of these, 12 patients are considered dropouts as they did not respond to the telephone interview; 5 listed for PLDD were treated surgically at another facility before completing conservative therapy. The analysis included the remaining 121 patients: average age 54.04 years (SD 13.69), ranging from 23 to 81 years. The group comprised 69 females (57%) and 52 males (43%).

The levels of pathology selected for treatment were as follows: L4–L5: 69.80%; L5–S1: 16.11%; L3–L4: 12.75%; L2–L3: 1.34%. Affected side: left, 51%; right, 47%; bilateral pain with median hernia, 2%. Three patients also had asymptomatic spondylolisthesis at a different level, two had prior spinal surgery at the same level, and one had previous hernia removal surgery at another level.

Baseline pain measured by VAS (retrospectively reported during interviews) was on average 7.36 (SD 1.43, range 5–10, median 7). Following the waiting period and conservative therapy (average 90 days, range 80–100 days), the average pain level was 7.06 (SD 1.66, range 2–10, median 7), representing an average reduction of 4.1% (Table 1 summarizes results according to VAS and Macnab criteria).

Eighteen patients (14.8%) declined PLDD surgery, satisfied with conservative therapy alone. In this subgroup, the average VAS before therapy was 6.6 (range 5–9, SD 1.08, median 7), and post-therapy was 4.72 (range 4–7, SD 1.6, median 5; average VAS reduction 28.5%); outcomes per Macnab criteria were 2 Excellent, 12 Good, 3 Fair, and 1 Poor. The characteristics of this group (age, sex, baseline VAS, and herniation level) were compared with those of the 103 patients who subsequently underwent PLDD. No statistically significant differences were observed between the two groups, although the baseline VAS tended to be lower in patients who were satisfied with conservative treatment alone, without reaching statistical significance (see Table 2).

Of the total patients, 103 underwent PLDD, with 70% treated at one level and 30% at two levels. Post-PLDD complications included local pain (4 patients), postoperative radiculitis (treated with corticosteroids, 5 patients), and one soft tissue hematoma at the needle entry site. Within 90 days post-PLDD, 8 patients required additional spinal surgery (6 microdiscectomies, 2 decompressions with fusion).

**Figure 2:**
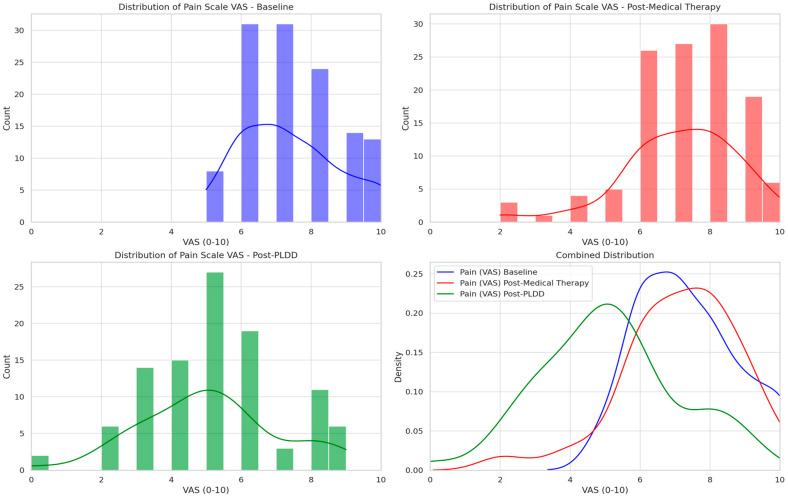
Displays patient-reported VAS scores at baseline, following medical therapy, and after PLDD. The histograms show the frequency of reported pain scores, overlaid with trend lines to indicate distribution shifts, while the combined density plot clarifies the overall trend in pain score modification across treatment stages.

Post-surgical therapy, the average pain level decreased to 5.11 (SD 1.98, range 0–9, median 5), representing an average VAS reduction of 30%. Figure 2 shows patient-reported VAS scores at baseline, after medical therapy, and following PLDD. The histograms detail the frequency of pain scores, with superimposed trend lines indicating distribution shifts. The combined density plot underscores the trend in pain modification across treatment stages. We analyzed the 18 patients who, satisfied with conservative therapy, chose not to proceed with PLDD. No significant differences were identified compared to the remaining patients; however, the sample sizes were too disparate to conduct a robust statistical analysis. We compared VAS results from baseline after conservative therapy (121 patients) and after PLDD (103 patients). The Wilcoxon test comparing baseline versus Post-conservative therapy indicated no significant difference in pain levels (*p* = 0.272). In contrast, a significant difference was found comparing baseline to post-PLDD therapy (*p* < 0.0001). The Mann–Whitney test confirmed significant differences in pain levels between post-medical and post-PLDD therapy (*p* < 0.0001).

**Figure 3:**
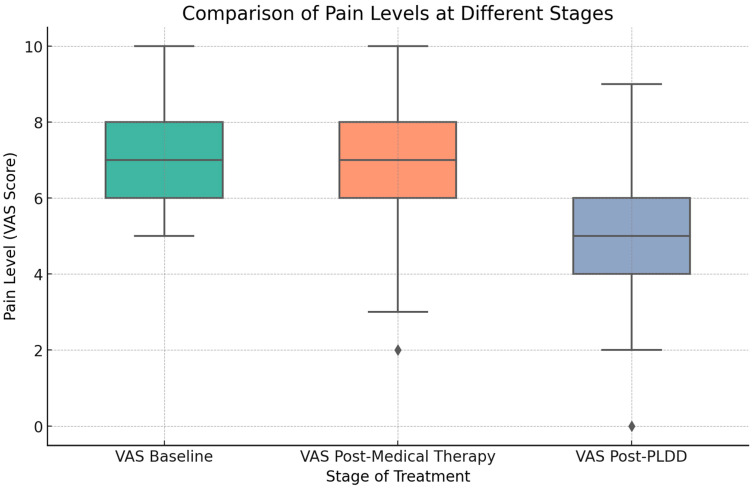
Pain level comparison. This boxplot illustrates the distribution of pain levels among patients at baseline, after medical therapy, and following PLDD surgical therapy. It displays medians, quartiles, and outliers, underscoring significant pain reduction post-surgery.

Figure 3 visually depicts the significant reduction in pain post-PLDD compared to baseline and post-medical therapy, showing the pain level distribution across the three assessment stages.

**Figure 4:**
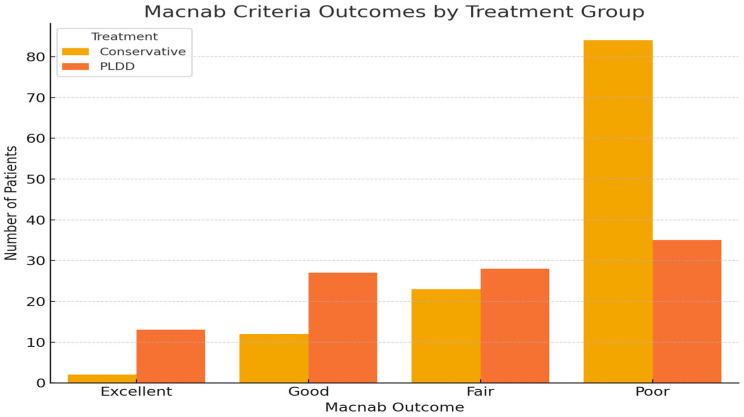
Comparison of treatment outcomes between medical therapy and PLDD surgery according to Macnab criteria. This graph delineates the differences in patient-reported outcomes between the two treatment modalities.

Results according to Macnab criteria (Figure 4 and Table 1): The descriptive analysis of the treatment outcomes, based on Macnab’s scale, for conservative therapy versus PLDD surgery is as follows:

- For conservative therapy:
  - Excellent: 2 patients (1.6%)
  - Good: 12 patients (9.8%)
  - Fair: 23 patients (18.9%)
  - Poor: 84 patients (69.4%)
- For PLDD surgery:
  - Excellent: 13 patients (12.6%)
  - Good: 27 patients (26.2%)
  - Fair: 28 patients (27.2%)
  - Poor: 35 patients (34.0%).

The distribution of outcomes indicates that patients who underwent PLDD surgery reported a better spread of results, with fewer patients rated as “Poor” and more patients achieving “Excellent”, “Good”, and “Fair” outcomes compared to the medical therapy group. These results were analyzed with a Chi-squared test (Figure 4): The Chi-squared test yielded a value of 33.27 with a *p*-value < 0.001 and three degrees of freedom. This significant *p*-value indicates a rejection of the null hypothesis (no significant differences), suggesting that the outcome distributions between the two treatment groups are different.

## 4. Discussion

Lumbar disc herniation (LDH) significantly affects global health, severely impacting patient quality of life [2,3] and adding substantial economic and social burdens. While initial treatment typically involves conservative methods integrating medical and physical therapies [3], established guidelines from various scientific societies also outline specific conditions that necessitate surgical intervention. Surgery is unanimously recommended for severe motor deficits, progressive neurological impairments, or cauda equina syndrome, especially when conservative treatments fail [1,2,10]. Accepted surgical techniques include microsurgical discectomy, tubular microscopic discectomy (also known as microendoscopic discectomy), and percutaneous endoscopic discectomy [1], providing targeted options when surgery is indicated.

However, managing patients for whom surgical intervention is not deemed appropriate remains challenging, especially those with contained disc herniations where surgical outcomes are often unsatisfactory [4,5]. Percutaneous discectomy, including percutaneous Laser disc decompression (PLDD), has long been considered a viable therapeutic option. The rationale behind PLDD is that the laser energy vaporizes a small portion of the nucleus pulposus within the disc, reducing its volume and relieving pressure on the surrounding nerve structures, thereby diminishing pain and improving function [8,9,11,12,13,14].

While some studies report long term benefits of PLDD, showing significant symptom relief over a two-year period [5,8,9,15], this view is not universally accepted. Other studies counter these positive outcomes by highlighting the inconclusive benefits and limited evidence supporting PLDD’s effectiveness, suggesting that its advantages may be overstated [4,6,16].

This polarity in findings illustrates the ongoing debate and the need for further rigorous studies to ascertain the true value of PLDD in clinical practice. In our series, PLDD was effective in reducing pain by an average of 30% compared to baseline (*p* < 0.001) and proved to be safe with a low complication rate. Importantly, we did not detect any major complications, aligning with findings from other studies [5,7,8,9,11,17]. However, it is crucial to note that our patient selection criteria focused on those with contained disc herniation, which likely influenced outcomes; thus, we do not consider PLDD a direct alternative to microdiscectomy.

Although some authors, notably Brouwer et al. [7,17], have proposed PLDD as an alternative to standard surgery, citing comparable one- and two-year outcomes to microdiscectomy, it is considered by the World Federation of Neurosurgical Societies (WFNS) as an intermediary between conservative therapy and surgery. Consequently, in our practice, we advocate using PLDD as an alternative to conservative therapy for patients with contained disc herniations for whom traditional surgery is not deemed appropriate based on existing guidelines. This strategy situates PLDD as an alternative therapy aimed at accelerating the healing process for patients, providing a quicker route to relief compared to conservative methods alone.

This placement underscores the need for direct comparisons between PLDD and conservative therapy, which are currently sparse in the literature; even in our center, we do not have two homogeneous groups of patients undergoing PLDD and conservative therapy. However, we have leveraged the waiting period prior to intervention: there is a notably lengthy waiting period for patients scheduled for PLDD, with a retrospective analysis revealing an average wait time of 90 days, ranging from 80 to 100 days. During this waiting period, all patients undergo standard conservative treatment (as detailed in our methodology section). We leveraged this waiting list to consider two phases of treatment as distinct groups: clinical outcomes following conservative treatment and outcomes following PLDD within the same cohort of patients. This approach allowed us to use the same patients for both interventions.

Although our study cannot be classified as a true case-control study, we view it as an observational comparative analysis of two treatments considered appropriate for the same patient demographic—those with contained disc herniation without neurological deficits due to compression. Therefore, the two groups are homogeneous, as they comprise the same patients. The treatments are somewhat comparable, as both are minimally invasive and partly reversible since even the minor complications post-PLDD are generally mild and reversible, such as radiculitis, local pain, and paravertebral muscle hematoma [9,16,18,19,20]. This setup provides a unique perspective on the relative efficacy of conservative therapy versus PLDD in managing contained lumbar disc herniation, reflecting real-world conditions where patients may experience both treatments sequentially.

Obviously it should be noticed that as all patients received conservative therapy prior to PLDD, the observed benefits of PLDD may partially reflect cumulative effects of both treatments. Although this sequential design reflects real-world clinical practice, it may limit our ability to isolate the independent efficacy of PLDD. This limitation should be taken into account when interpreting the results; moreover, the exclusion of 18 patients who responded well to conservative treatment may have introduced a selection bias, potentially overestimating the benefit of PLDD among patients who remained symptomatic. However, retrospective analysis showed no statistically significant differences in baseline VAS scores or demographics between the two groups, although the sample size of the conservative-only group was limited (Table 2).

The primary outcome of our study showed that while the average baseline Visual Analog Scale (VAS) score was 7.36, it only modestly decreased to 7.06 following conservative therapy, a reduction of 4.1%. In contrast, after PLDD, the average pain level significantly dropped to 5.11, marking a 30% reduction from baseline (*p* < 0.0001). This stark improvement significantly outperformed the conservative approach, as evidenced also by the Macnab criteria, which showed a substantial increase in ‘Excellent’ and ‘Good’ ratings (Chi-squared *p* < 0.001). These findings are supported by other research [5,15,21] indicating major pain score reductions and functional improvements in PLDD patients, enhancing quality of life and functional status over conservative methods alone. For example, Hashemi et al. [8]. observed notable enhancements in mobility and daily activities over two years, reinforcing the efficacy of PLDD in clinical practice.

In our analysis, it is notable that 18 patients satisfied with medical therapy opted out of progressing to PLDD, indicating that a minor segment achieved symptom resolution through conservative measures alone. This outcome is significantly lower than broader literature expectations, where 70–80% of patients reportedly achieve near-complete symptom alleviation with conservative therapy [1,2,3]. This variance might be attributed to non-strict adherence to the prescribed conservative protocols among our study participants. It is possible that some patients found the conservative treatment regimen—comprising rest, medication, and physical therapy—more burdensome and thus were less diligent in following it, preferring to wait for the less demanding PLDD procedure. Such discrepancies are crucial for assessing the relative effectiveness of treatment methodologies.

Despite these methodological limitations, PLDD has proven more effective than conservative therapy in our experience, offering rapid results that can provide clinical and economic benefits, especially considering the high costs of extended conservative treatments. Focused on short term outcomes within a six-month period, our analysis supports PLDD as a viable early intervention, potentially accelerating symptomatic relief compared to the traditional waiting period for conservative treatment results. However, the long term effects of PLDD remain to be fully evaluated, necessitating additional studies. According to van den Akker-van Marle et al. [22], PLDD is also more cost-effective than conventional surgery in the first year, although its cost-effectiveness varies with different economic thresholds. The comparative efficacy of PLDD, a minimally invasive procedure, against conventional medical therapy underscores the evolving landscape of lumbar disc herniation treatment strategies. While conservative measures remain the first line of treatment for a majority of patients, the subset experiencing intractable pain or not amenable to surgical intervention poses a therapeutic challenge. In this context, PLDD emerges as a promising alternative, potentially bridging the gap between non-invasive and surgical modalities. This positioning enhances PLDD’s appeal as a transitional therapy, offering a quicker, effective response to pain relief compared to conventional methods alone. Minimally invasive surgical techniques are becoming more common in neurosurgery due to their potential to lower risks and improve patient outcomes [23,24,25]. Consistent with this trend, our findings support the use of minimally invasive spine interventions like PLDD, which reduce morbidity and speed up recovery. The success of PLDD, evidenced by a low complication rate and aligned with previous studies [6,8,9,11], depends critically on precise patient selection [5,6,9]. Our study’s inclusion and exclusion criteria reflect this need for careful patient screening to maximize the benefits of PLDD and ensure optimal clinical results.

In our experience, we have used the ARGO diode laser probe, with a total of 500 joules over 40 s (divided into 250 joules at the center of the disc and 250 joules at the posterolateral edge). The literature indicates that there is no consensus on the optimal laser technology and parameters to use for percutaneous laser disc decompression (PLDD), as highlighted by Gazzeri et al. [5]. Some researchers have explored the use of neuronavigation, US- or CT-guidance, and robotic arms to enhance precision [26,27,28]. However, current evidence does not show significant differences in outcomes based on the equipment or technique employed. In our practice, we employ a free-hand technique with 2D intraoperative fluoroscopy, supported by preoperative MRI and CT scans for planning the trajectory, particularly in cases involving the L5–S1 level where the iliac crest may pose a challenge in accessing the disc.

### Limitations

Despite the encouraging outcomes, the limitations inherent to the study’s retrospective design and the absence of a randomized control group warrant cautious interpretation of the results. It is important to consider that the conservative treatment was administered prior to PLDD, potentially influencing the favorable outcomes observed with PLDD. This sequence might suggest that the initial conservative therapy could have partially contributed to the positive effects seen with the subsequent PLDD intervention. Therefore, the results, while promising, necessitate further investigation through prospective, randomized studies to disentangle the individual contributions of each treatment modality and establish definitive clinical guidelines for PLDD in lumbar disc herniation treatment.

Another limitation of our study is the retrospective assessment of outcomes through telephone interviews. This approach may have introduced recall bias, particularly for subjective measures such as pain intensity and functional status, and should be considered when interpreting the findings.

Finally, another limitation in our study that should be mentioned is the relatively low rate of success with conservative therapy in our study (14.8%) which contrasts with higher rates (up to 70–80%) reported in the literature. This discrepancy may reflect strict inclusion criteria, the referral of more refractory cases to our center, or potential variations in adherence to conservative protocols. Such factors may explain the reduced effectiveness of conservative therapy observed in our cohort in favor of PLDD.

## 5. Conclusions

Our study shows that PLDD significantly reduces pain and improves functional outcomes in patients with contained lumbar disc herniation, offering a valid alternative to conservative therapy. Specifically, PLDD led to a 30% reduction in pain scores, markedly higher than the modest 4.1% decrease observed with conservative treatment alone. These results support the efficacy of PLDD in providing rapid clinical improvement in the short term. However, it is important to acknowledge the limitations of our study, including its retrospective design and the lack of a randomized control group, which may affect the generalizability of the findings. Despite these drawbacks, given its capacity to quickly alleviate symptoms, PLDD should be considered at the initial assessment for patients with contained disc herniation, potentially expediting relief and enhancing quality of life. Future prospective, randomized trials are necessary to confirm these findings and refine clinical guidelines for the use of PLDD in managing lumbar disc herniation.

## Figures and Tables

**Table 1:** Results according to VAS and Macnab criteria for both treatments.

		VAS				Macnabb Criteria			
	N°	Average	Range	SD	Median	Excellent	Good	Fair	Poor
Baseline	121	7.36	5–10	1.43	7				
Post-conservative treatment	121	7.06	2–10	1.66	7	2 pts (1.6%)	12 pts (9.8%)	23 pts (18.9%)	84 pts (69.4%)
Post-PLDD	103	5.11	0–9	1.98	5	13 pts (12.6%)	27 pts (26.2%)	28 pts (27.2%)	35 pts (34%)

**Table 2:** Baseline characteristic of the conservative group versus the PLDD group.

	Mean Age (Years)	Sex (M/F)	VAS Baseline
Conservative only (n = 18)	52.39 (SD 15.33)	M 38.8% F 61.2%	6.6 (range 5–10, SD 1.08)
PLDD group (n = 103)	54.39 (SD 13.42)	M 43.7% F 56.3%	7.1 (range 6–10, SD 1.65)

## Data Availability

No new data were created or analyzed in this study.

## Abbreviations

The following abbreviations are used in this manuscript:

LDH	lumbar disk herniation
PLDD	percutaneous laser disk decompression
VAS	visual analog scale
SD	Standard deviation
STROBE	Strengthening the Reporting of Observational Studies in Epidemiology
